# Stability of Phase Relationships While Coordinating Arm Reaches with Whole Body Motion

**DOI:** 10.1371/journal.pone.0146231

**Published:** 2015-12-31

**Authors:** Romy S. Bakker, Luc P. J. Selen, W. Pieter Medendorp

**Affiliations:** Radboud University Nijmegen, Donders Centre for Cognition, Donders Institute for Brain, Cognition and Behaviour, Nijmegen, The Netherlands; University of California, Merced, UNITED STATES

## Abstract

The human movement repertoire is characterized by the smooth coordination of several body parts, including arm movements and whole body motion. The neural control of this coordination is quite complex because the various body parts have their own kinematic and dynamic properties. Behavioral inferences about the neural solution to the coordination problem could be obtained by examining the emerging phase relationship and its stability. Here, we studied the phase relationships that characterize the coordination of arm-reaching movements with passively-induced whole-body motion. Participants were laterally translated using a vestibular chair that oscillated at a fixed frequency of 0.83 Hz. They were instructed to reach between two targets that were aligned either parallel or orthogonal to the whole body motion. During the first cycles of body motion, a metronome entrained either an in-phase or an anti-phase relationship between hand and body motion, which was released at later cycles to test phase stability. Results suggest that inertial forces play an important role when coordinating reaches with cyclic whole-body motion. For parallel reaches, we found a stable in-phase and an unstable anti-phase relationship. When the latter was imposed, it readily transitioned or drifted back toward an in-phase relationship at cycles without metronomic entrainment. For orthogonal reaches, we did not find a clear difference in stability between in-phase and anti-phase relationships. Computer simulations further show that cost models that minimize energy expenditure (i.e. net torques) or endpoint variance of the reach cannot fully explain the observed coordination patterns. We discuss how predictive control and impedance control processes could be considered important mechanisms underlying the rhythmic coordination of arm reaches and body motion.

## Introduction

Suppose you are standing in a bus that suddenly speeds up. To prevent yourself from falling, you quickly reach for the safety rail and stabilize your body. The ability to reach in such an accelerating environment is neurally quite demanding, requiring an accurate estimate of the position and dynamics of the arm and the body, as well as the location of the rail that is fixed relative to the bus. The objective of the present study is to understand the mechanisms that underlie the estimation and the subsequent action decisions when the body accelerates.

Previous studies mainly focused on the effect of body motion on reaches to world-fixed targets [1–5]. In this case, compensation for body acceleration is thought to occur through vestibular-mediated manual reflexes that control the reach [1,2,6], analogous to the vestibular ocular reflexes that maintain gaze stability in space [7].

When targets are fixed to the body the vestibular-mediated reflexes should be suppressed. It should be realized, however, that this does not simplify the control problem to generating reaches towards world-fixed targets while the body is stationary. Even when manual reflexes are switched off, inertial forces due to body motion will still perturb the reach [8]. To generate the reaching movement, the brain needs to compute a control policy [9] that takes into account the inertial forces induced by the body motion.

Temporal coordination is an important component of this control policy. While in some situations the reach and body movement are simultaneously initiated, in other cases the reach is initiated while the body is already in motion [4]. How does the brain decide when to commence the reach when the body is in motion?

Here, we test the brain’s evaluation of limb and body dynamics when coordinating body-referenced reaching movements with passively induced sinusoidal body-motion. It could be argued that the brain anticipates the current and future induced (inertial) forces, based on vestibular inputs [10]. From these predictions, the brain can build a control policy that could be considered as the outcome of a coupled dynamical system of three rigid bodies with different biomechanical properties; the upper arm and forearm that need to be coordinated with the cyclic motion of the trunk [11]. Such systems typically express themselves through synergies [3,12,13], selected based on cost functions [14] and neural constraints [15,16].

In the present study, subjects had to continuously reach between two body-fixed targets, positioned either orthogonal or parallel to the direction of the body motion. We hypothesize that the phase relationship that develops in the coordination of arm and body motion is a reflection of a particular cost function that is minimized [17,18].

We considered two cost functions: one based on energy expenditure [19,20] and the other based on endpoint accuracy [21]. Because the inertial forces on the limb differ for the two reach directions, the phase related costs would also differ. As a result, we predict differences in the stability of the phase relationships for the orthogonal and parallel conditions. In the current paper, we express stability in terms of the preservation over time of an imposed phase relationship between the arm and whole body motion.

Our experimental results suggest that the brain takes the inertial forces into account when coordinating reach and body motion. We further find clear differences between in-phase and anti-phase stability in the two reach conditions, although we cannot entirely explain these by optimization criteria such as energy costs or endpoint accuracy.

## Material and Methods

### Participants

Ten right-handed subjects (4 male, 6 female aged 20–26 years) gave their written consent prior to participating in the experiment. All subjects had normal or corrected-to-normal vision and had no known motor deficits. The study was approved by the ethics committee of faculty of social sciences of Radboud University.

### Setup

**Fig 1:**
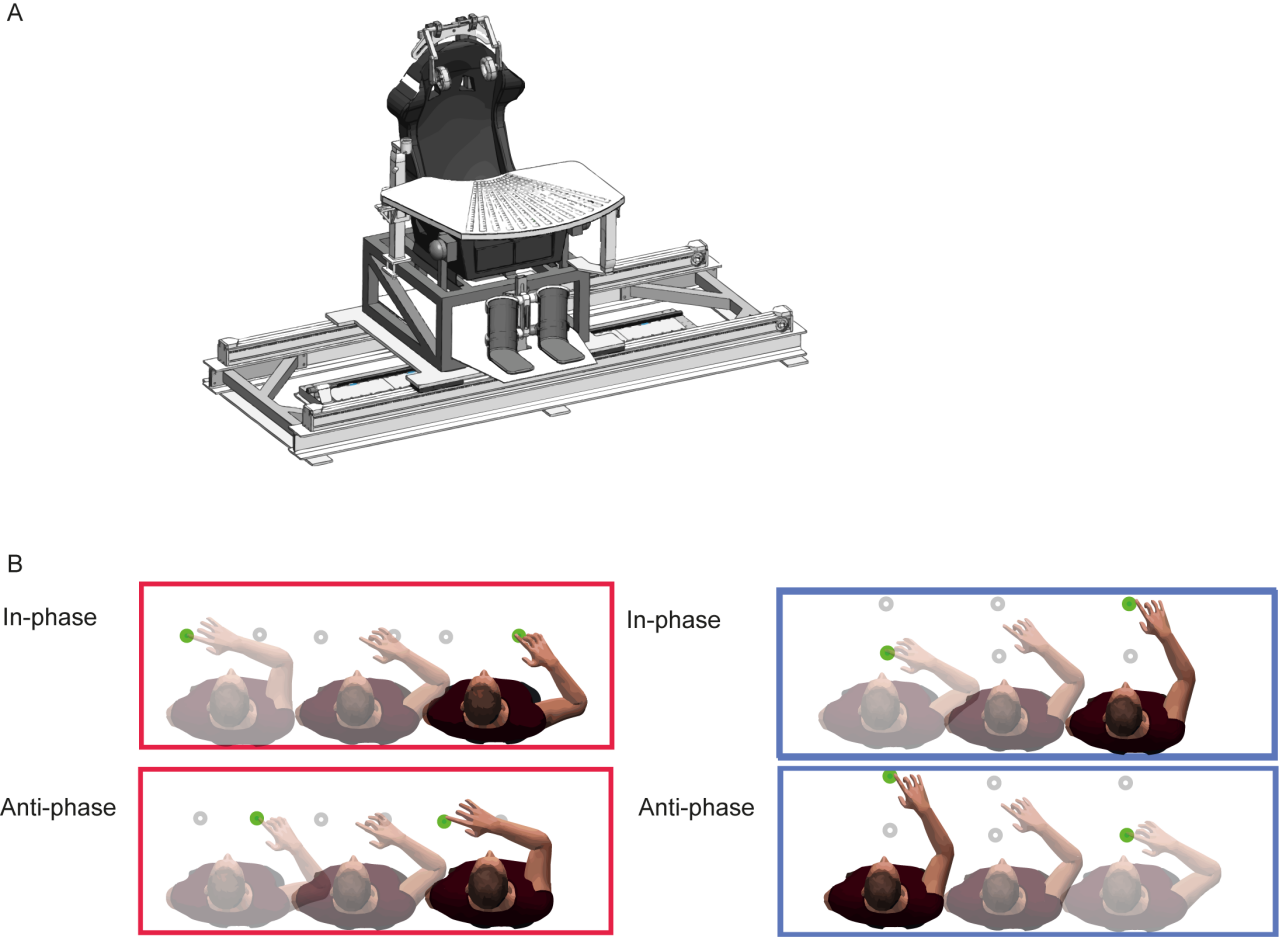
Experimental setup and paradigms. (A) Subjects were seated on a linear sled and performed right hand reaching movements to visual targets presented on a sled mounted table. (B) Parallel reaching. (top) in-phase, (bottom) anti-phase. (C) Orthogonal reaching. (top) in-phase, (bottom) anti-phase.

Subjects were translated on linear sled (Fig 1A). The sled, powered by a linear motor (TB15N, Technotion, Almelo, The Netherlands), was controlled by a Kollmorgen S700 drive (Danaher, Washington, DC) with accuracy better than 0.034 mm, 2 mm/s, and 150 mm/s^2^. During the experiment, the sled moved sinusoidally at a fixed frequency of 0.8 Hz over 30 cm with a maximum acceleration of 4.1 m/s^2^. Subjects were seated with their interaural axis aligned with the direction of the sled motion. They were restrained with a five-point seat belt and the head was firmly fixed with an ear-fixed mold and a chin rest. Auditory stimuli were presented using in-ear headphones. Emergency buttons at either side of the chair could immediately stop the sled motion, if needed.

Targets were presented by green and red LEDs, integrated into a table, mounted on the sled in front of the subjects at the level of their abdomen (Fig 1A). The LED in front of the center of the body, at a distance of 20 cm, corresponded to the origin of our body-fixed coordinate system, in which the x-axis was defined orthogonal and the y-axis aligned to the direction of body motion. The parallel targets were positioned at a distance of 20 cm from the subjects’ body, both at 15 cm from the body midline. The targets positioned orthogonal to the whole body, were both placed at the body midline, at +10 cm and +40 cm distance from the subject.

The position of the sled and the position of the tip of the right index finger were recorded in real-time at 100 Hz using an Optotrak Certus system (NDI, Northern Digital Instruments, Waterloo, Canada). The experiment was performed in a completely dark room, except for the target LEDs. In addition, a small white LED was attached to the right index finger of the subject, which allowed subjects to see their hand during the reach. The experiment and the setup were controlled using software written in Python.

### Experimental paradigm

Before the body motion started, subjects positioned their fingertip at the location of the central LED (the origin). While in motion, subjects were instructed to reach between two body-fixed targets, touching each target once per cycle of sled motion The two targets defined parallel reaching movements (Fig 1B) or orthogonal reaching movements (Fig 1C) relative to sled motion. Each condition was tested in separate blocks, consisting of about 40 cycles with two reaches per cycle (one reach to both targets).

During the first 16 cycles, a metronome entrained the phase relationship between arm and sled motion. This metronome instructed either an in-phase or an anti-phase relationship by both visual and auditory guidance of the movements. The auditory guidance consisted of two beeps (duration = 0.1 s) that differed in pitch, presented at the turning points of the sled motion. The visual assistance entailed that a target turned on at the midpoint of the sinusoidal sled motion, lasting for half a cycle, such that subjects only saw one target at a time. After 16 cycles, the metronome was switched off. During the following motion cycles, white noise was presented through the in-ear headphones and both targets were illuminated permanently. A reach was considered a hit when the finger reached within a radius of 5cm from the target. After 48 hits, the targets were turned off and the sled was stopped. Then, the next block started.

In the parallel condition, the phase relationship between finger and body motion is unambiguously defined (Fig 1B). For the orthogonal condition we defined arm movement away from the body as in-phase with rightward body motion (Fig 1C). Subjects performed alternating series of four blocks, in which either the orthogonal or parallel condition was tested, counterbalanced across subjects. In total, a minimum of 12 blocks per condition was tested. Imposed in-phase and anti-phase relationships were randomized across blocks. Prior to the actual data collection, subjects performed a few practice reaches to familiarize with the sled motion and reaching to the body-referenced targets.

### Data analyses

Data analyses were performed offline with Matlab 2013a (Mathworks). Positions of the sled and the tip of the right index finger were recorded at 100 Hz. Spline interpolation was used to reconstruct missing data points due to obstruction of the Optotrak markers. Velocity time series were calculated from the position time series by taking the gradient. Position and velocity time series were used to calculate the phase φ of the finger and the body motion for each block according to:

$$\varphi_{sled-}\varphi_{finger}\;=\;{\text{tan}}^{-1}\;\left(\frac{v_{sled}\left(t\right)}{x_{sled}\left(t\right)}\right)-{\text{tan}}^{-1}\;\left(\frac{v_{finger}\left(t\right)}{x_{finger}\left(t\right)}\right)+C$$ (1)

Here, *φ*_*sled*_ represents the phase of the sled and *φ*_*finger*_ the phase of the finger at each sample. Finger position *x*_*finger*_ and sled position *x*_*sled*_, as well as the finger velocity *v*_*finger*_ and sled velocity *v*_*sled*_ were each normalized to the range [–1,1] for each block, by dividing it by its maximum value [22].

Phase was calculated for three different parts of a block: the last 8 motion cycles of the metronome-guided part (*metronome on*), the first half without metronome guidance (*early off*) and the final half without metronome guidance (*late off*). Blocks were accepted for further analyses based on the *metronome on* data: In-phase blocks were accepted if more than 90% of the samples had a phase that was between -90 and +90 deg. Anti-phase blocks were accepted if more than 90% of the phase data were between 90 and 270 deg. Based on these criteria, three subjects were excluded from the anti-phase parallel condition, of which two were also excluded from the anti-phase orthogonal condition. To analyze the number of phase transitions, we looked at the changes of relative phase after the metronome had been turned off. We counted a phase transition when the relative phase shift was larger than 100 degrees from the imposed phase relationship and this new phase relationship was maintained for at least four seconds. The latter constraint was added to avoid interpreting random phase wandering as phase transitions. We counted the number of blocks that contained a phase transition and used bootstrapping to calculate a mean and standard deviation across subjects.

Statistics were performed over the three different experimental parts *metronome on*, *early off* and *late off* and over four different conditions: *parallel in-phase*, *parallel anti-phase*, *orthogonal in-phase*, *and orthogonal anti-phase*. Means and standard deviations of the relative phases were calculated using circular statistics [23]. Standard one-sample t-tests were performed to test whether phase relationships were at perfect in-phase 0° or at perfect anti-phase 180°. Second, regular paired sample t-test were performed to test for significant differences in relative phase over time and between conditions.

### Model

**Fig 2:**
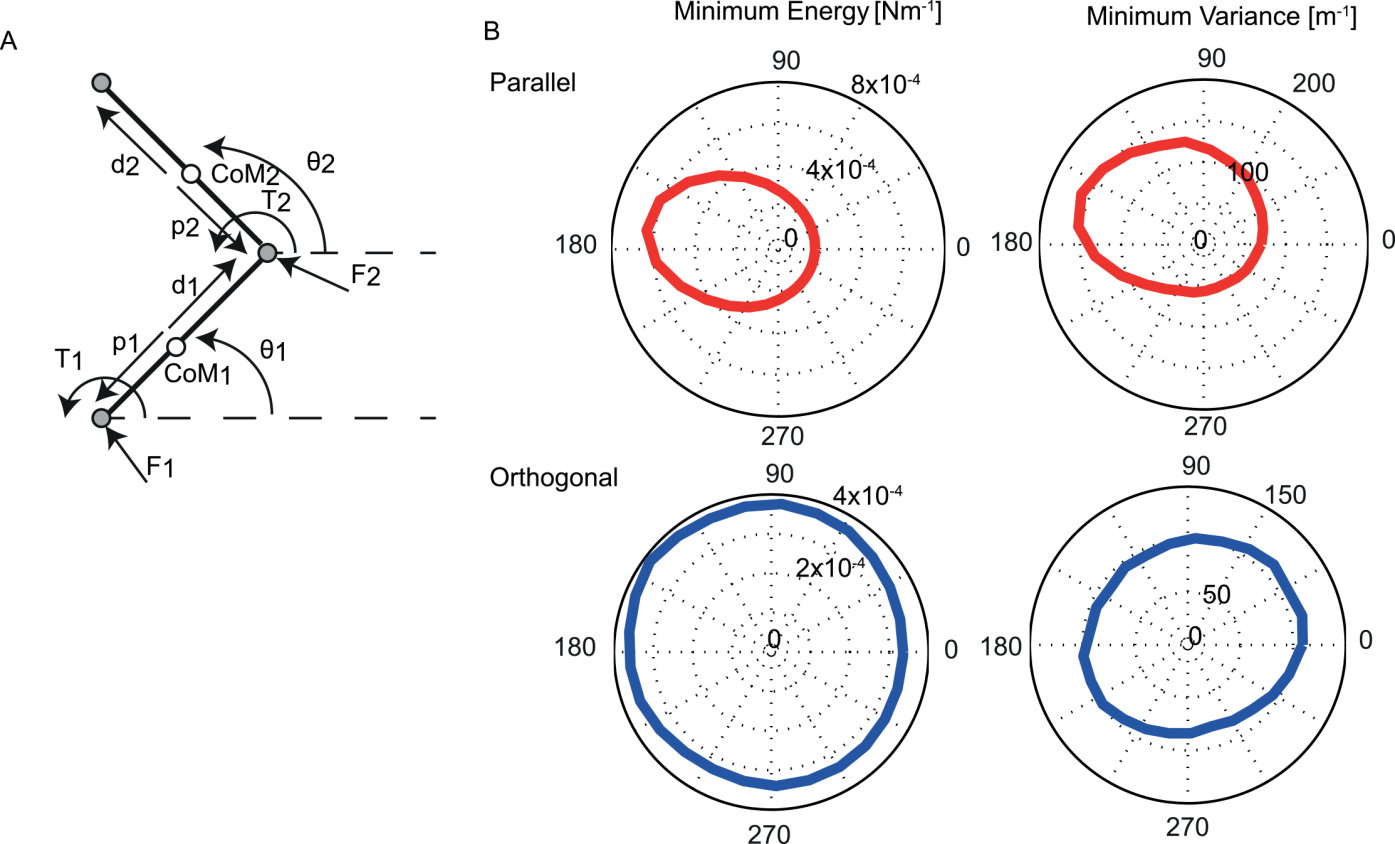
The 2-link model and predictions. Diagram of the 2-link arm model (B) Costs based on the minimum energy model. Cost is expressed as the inverse of the summed joint torques for the different phase angles between hand and whole body motion (left); Costs based on the minimum end-point model. Cost is expressed in terms of end-point variance for the different phase angles between hand and whole body motion (right).

We investigated whether biomechanical cost factors, either energy expenditure or endpoint accuracy, underlie the coordination patterns between arm movements and whole body motion. If the coordination were driven by energetic costs, we would expect subjects to show a more stable phase relationship around the minimum energy solution. If arm-trunk coordination were driven by the minimization of endpoint variance, we would expect subjects to show more stability for the phase relationship minimizing endpoint variance. To make predictions about the phase relationships, we built a generic planar arm model (Fig 2A), by which reaching under whole body motion was simulated. The simulation provided estimates of the energetic costs and the endpoint variance associated with the different phase relationships between arm and whole body motion. From the estimates, we derived the ‘optimal’ phase relationship.

The origin of the generic planar arm model was positioned at the shoulder, which accelerated due to the passively imposed whole-body motion. Parameters of the model arm were based on previous literature (Table 1) [24]. Parameters $\vec{p}$ and $\vec{d}$ represent the distance of the proximal and distal joint from the Center of Mass (CoM) of either of the two links, respectively. The inertia (I_1_ and I_2_) are defined relative to the CoM. Targets were positioned as in the actual experiment.

**Table 1:** Parameters of the 2-link arm. Note that *k* and *b* are rotational stiffness and damping.

Parameter	Value
$\left|\vec{p_{1}}\right|$	0.12 (m)
$\left|\vec{p_{2}}\right|$	0.20 (m)
$\left|\vec{d_{1}}\right|$	0.13 (m)
$\left|\vec{d_{2}}\right|$	0.15 (m)
m_1_	1.93 (kg)
m_2_	1.52 (kg)
I_1_	0.04 (Nms^2^)
I_2_	0.06 (Nms^2^)
b	-0.2 (Nms)
k	-10 (Nm)

The model was used to simulate reaches parallel and orthogonal to the whole body motion. The movement of the hand in body-centered coordinates in either the x-direction (parallel reaches) or y-direction (orthogonal reaches) during a full body motion cycle was defined by the concatenation of two minimum jerk trajectories [25]. Using inverse kinematics, we derived the corresponding joint angles, joint angular velocities and joint angular accelerations, as well as the Cartesian motion of the centers-of-mass of the limb segments. For the latter, the kinematics of the shoulder motion was also taken into account.

From the time traces of the kinematics we calculated the required torques using inverse dynamics. The Newton-Euler equations of motion for the two individual links (upper arm and forearm) follow in a straightforward manner. The arm moves relative to the body motion. Because we consider the arm motion in the horizontal plane, the effects of gravity can be neglected. Movement equations are defined relative to the center of mass (CoM). The Newton-Euler equations for link 1 (upper arm) are given by:

$${\vec{F}}_{1}-{\vec{F}}_{2}=\;m_{1}({\vec{a}}_{1}+{\vec{a}}_{s})$$ (2)

$$I_{1}\alpha_{1}={\vec{p}}_{1}\times {\vec{F}}_{1}-{\vec{d}}_{1}\times {\vec{F}}_{2}+\tau_{1}-\tau_{2}+b\omega_{1}-b\omega_{2}$$ (3)

For link 2 (forearm):

$${\vec{F}}_{2}=m_{2}({\vec{a}}_{2}+{\vec{a}}_{s})$$ (4)

$$I_{2}\alpha_{2}={\vec{p}}_{2}\times {\vec{F}}_{2}+\tau_{2}+b\omega_{2}$$ (5)

The forces generated through the passive motion of the body and arm are represented by ${\vec{F}}_{1}$ for the shoulder and ${\vec{F}}_{2}$ for the elbow. τ_1_ represents the active shoulder torques and τ_2_ the active elbow torques. The terms $\vec{a_{1}}$ and $\vec{a_{2}}$ represent the Cartesian accelerations of the center of mass of the upper arm and the lower arm relative to the shoulder. These were calculated based on joint angles, angular velocities and angular accelerations from the minimum jerk trajectories. For the calculation of the torques, a damping term was added to the joint velocity (i.e. bω, Table 1) to prevent the arm from making impossible movements. Sinusoidal whole body acceleration, which is represented at the shoulder joint by a_s_, varies only along the x-direction:

$${\vec{a}}_{s=}\left[\begin{array}{c} -\frac{4\pi^{2}}{{\left(T\right)}^{2}}A.\text{cos}\;\left(\frac{2\pi t}{T}+phase\right)\\ 0 \end{array}\right]$$ (6)

in which *T* is the period time, *A* the amplitude and *t* is the time. *Phase* represents the 26 different phase relationships between the arm and the whole body motion, equally distributed between 0 and 2π. The system of movement equations was solved simultaneously.

#### Cost in terms of energy

The integral of the squared joint torques over a full movement cycle of the individual joints was used as an estimate of the energy consumption. Next, we expressed those energy estimates as their inverses to make them comparable to the occurrence of certain phase relationships in the experimental data. These inverse energy estimates showed a peak around 170° anti-phase for the parallel reaches (Fig 2B). The peak of shoulder and elbow was similar in phase but differed in magnitude (higher for shoulder than elbow). For the orthogonal reaches, the model did not show a clear energetic optimum across the relative phases (Fig 2B).

#### Cost in terms of endpoint variance

In order to estimate the accuracy associated with different phase relationships between arm and body motion, the joint torques resulting from the inverse kinematics and dynamics were contaminated with signal-dependent noise [21] and subsequently used in a forward simulation. For simulating the forward dynamics the movement equations were re-ordered to make the torques, as derived from the inverse dynamics, contaminated with signal-dependent noise. By rewriting the movement equations, the joint accelerations could be calculated. A damping term and a stiffness term were added to the joint velocities and angles (Table 1). Using Newton-Euler integration, the joint angular velocities and joint angular accelerations were converted into joint positions. The simulation was run 2000 times, each with independently drawn noise, for the 26 phase relationships. From these 2000 simulations we estimated the angular accelerations, velocities and positions of the arm. From the joint angles, endpoint distributions in Cartesian coordinates were calculated. For the parallel reaches, the minimum variance model showed an optimal relative phase around 160°, which is in-phase (Fig 2B). For the orthogonal reaches, the model did not show a clear optimal relative phase (Fig 2B).

## Results

Subjects made reaching movements parallel and orthogonal to their passively-induced sinusoidal body motion. A metronome first entrained the reaching movements to be in-phase or anti-phase with the body movement. After this guidance was switched off, subjects had to keep making continuous reaching movements without any constraint on the phase relationship.

### Movement traces

**Fig 3:**
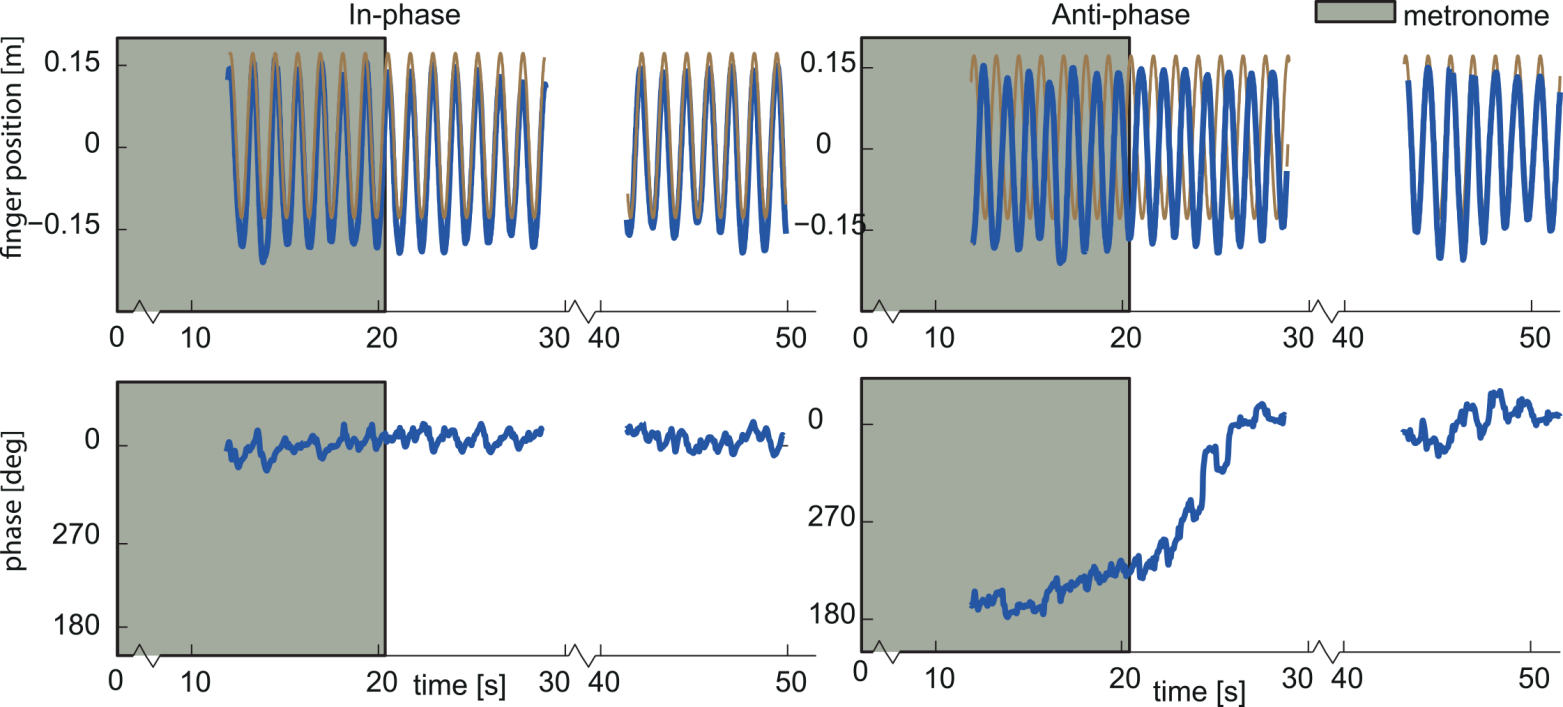
Single trial data one representative subject in the parallel condition. (Top) Finger kinematics in body-centered coordinates (blue). Sled motion in world-centered coordinates (brown) for in-phase entrainment (left) and anti-phase entrainment (right). (Bottom) Relative phase between finger and sled movement for in-phase (left) and anti-phase (right). The grey area on each plot represents the first 16 cycles, during which timing of hand motion was guided by a metronome.

Fig 3 shows the position of the finger and the body as a function of time for one representative subject in the parallel condition, for both the in-phase and anti-phase entrainment. Note that the finger motion is represented in body coordinates. The body moved sinusoidally at a frequency of 0.83 Hz and 15cm amplitude (as shown in brown in Fig 3). Hand motion, shown in blue, was guided by a metronome for the first 16 cycles, either in-phase or anti-phase with the body motion. When the metronome was turned off, the entrained hand motion continued during the first cycles in both conditions. The in-phase relationship appeared stable throughout the block, while the anti-phase relationship drifted and showed several transitions to an in-phase relation. We analyzed the number of phase transitions per condition. In the parallel condition, subjects made phase transitions in 13% (σ = 4%) of the in-phase blocks and in 26% (σ = 7%) of the anti-phase blocks. In the orthogonal condition, phase transitions were visible in 27% (σ = 6%) of the in-phase blocks and in 32% (σ = 6%) of the anti-phase blocks. In the following paragraphs, we will focus on this entrainment-dependent drift.

### Reaches parallel to body-motion

**Fig 4:**
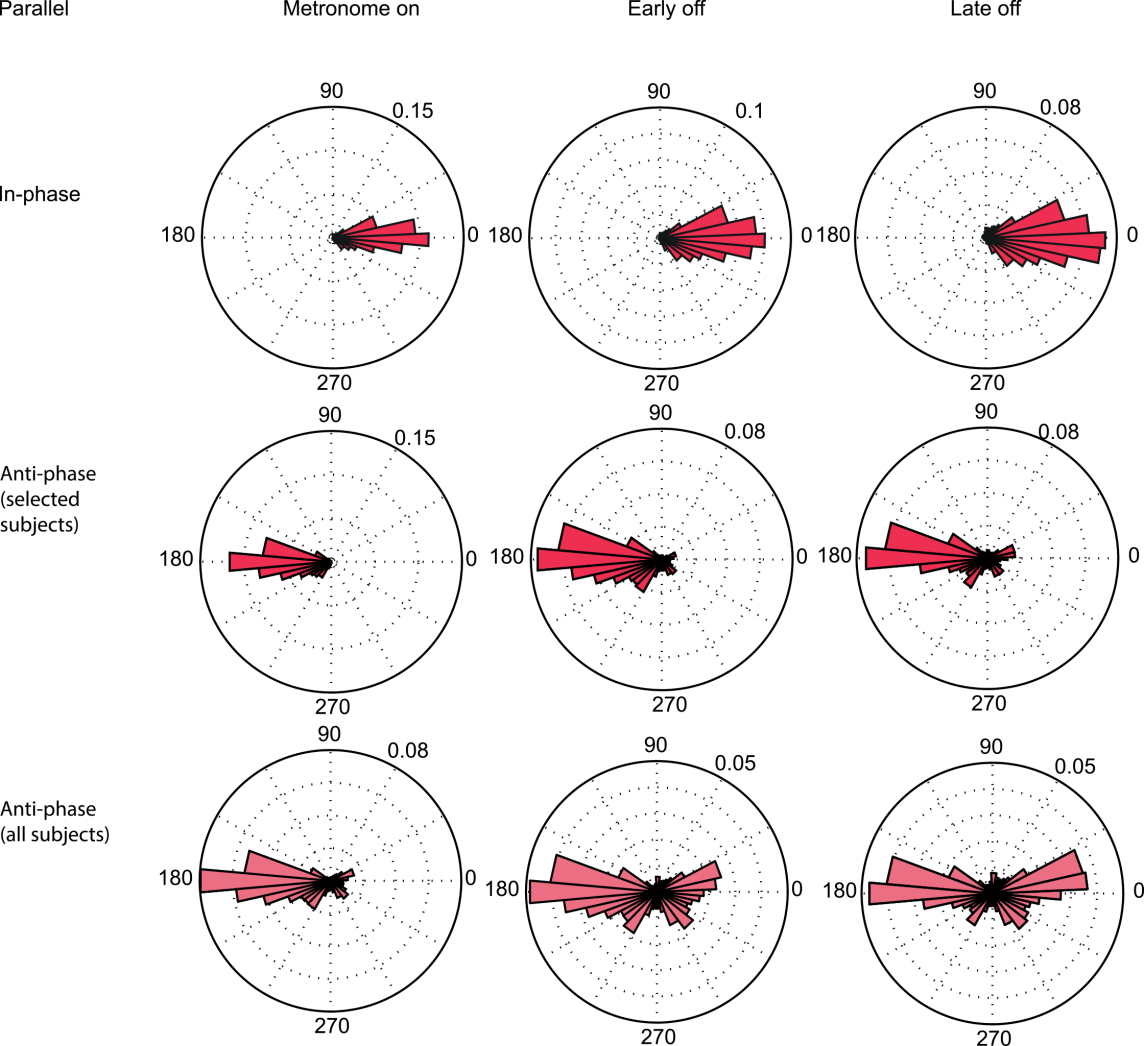
Distribution of relative phase of all subjects in the Parallel condition. (Top) In-phase entrainment. (Middle) Anti-phase entrainment of selected subjects. (Bottom) Anti-phase entrainment of all subjects.

Fig 4 shows the distribution of the relative phases in the parallel condition, across all 10 subjects. The top panel presents the block in which initially an in-phase relationship was instructed. Indeed, the average phase during *metronome on* was in-phase (μ = 357.5°, σ = 15.5°), which was not significantly different from a perfect in-phase angle of 0° (t(9) = -0.55, p = 0.6). Without the entraining metronome, the mean relative phase did not change and was not dissociable from 0° (t(9) = 0.14, p = 0.89), although it tended to become more variable (t(9) = -2.21, p = 0.055).

The second row of Fig 4 shows the blocks that started with an anti-phase entrainment. Only 7 of 10 subjects were able to control their hand motion according to this relationship, albeit not perfectly at 180° (μ = 192.7°, σ = 19.1°)(t (6) = 2.6, p = 0.04). Without metronome guidance the movement coordination became much less stable. This is shown by a significant increase in variance for the *early off* compared to *metronome on* (t2) (t(6) = -2.93, p = 0.026), as well as for the *late off* compared to the *metronome on* (t3) (t(6) = -2.67, p = 0.037). This increase in variance was accompanied by phase transitions toward an in-phase relation.

The instability of the anti-phase coordination pattern becomes even more pronounced when including the initially excluded 3 subjects (Fig 4, bottom panel). This clearly indicates that it was harder for subjects to coordinate their reaches in anti-phase with the whole body motion, compared to the in-phase relationship in the parallel condition.

### Reaches orthogonal to body motion

**Fig 5:**
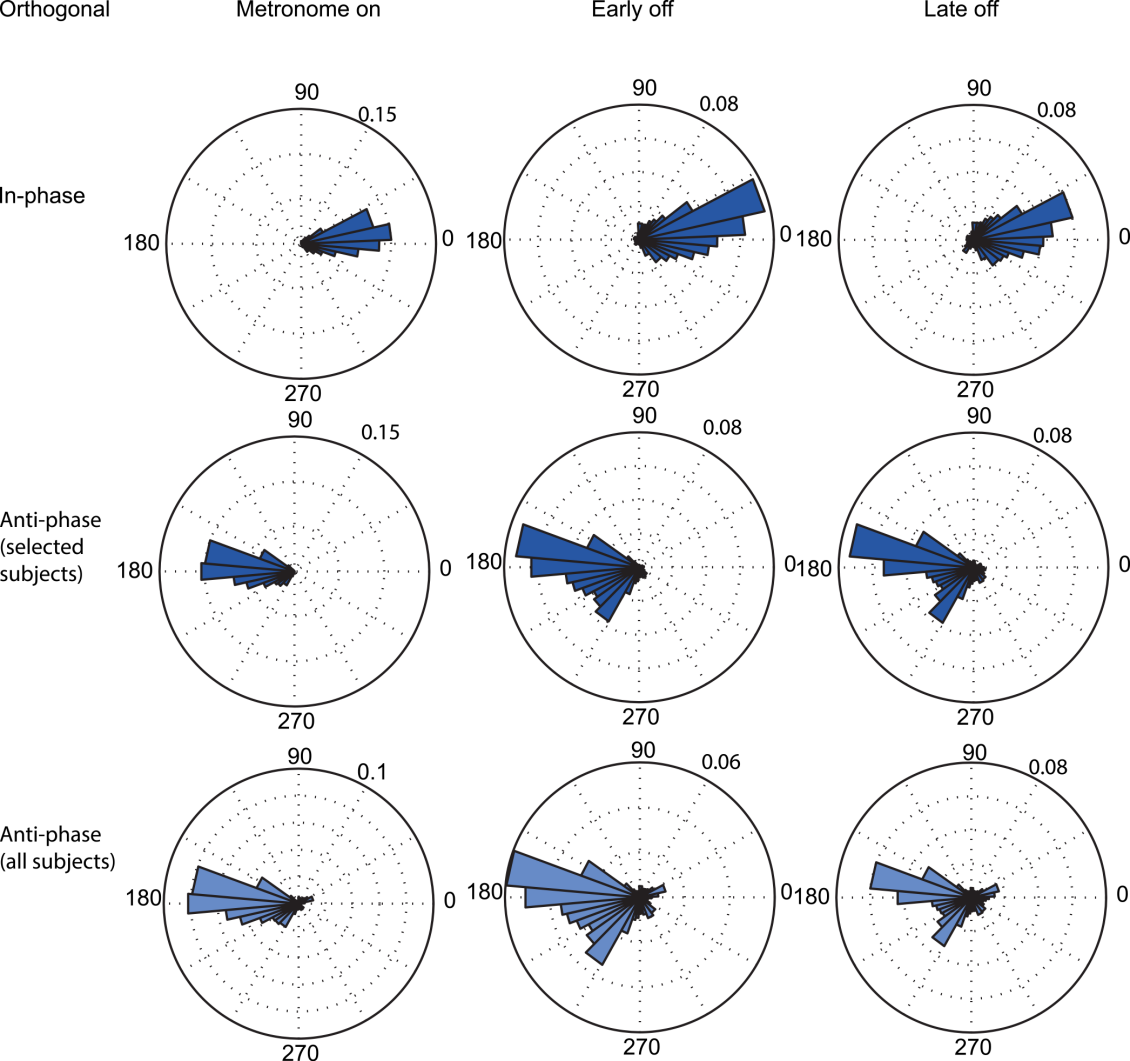
Distribution of relative phase of all subjects in the Orthogonal condition. (Top) In-phase entrainment. (Middle) Anti-phase entrainment of selected subjects. (Bottom) Anti-phase entrainment of all subjects.

Fig 5 shows the distributions of the relative phases in the orthogonal condition. In-phase hand motion, defined as reaches away from a rightward moving body, was fairly stable and within the required range (μ = 4.1°, σ = 19.4°) (Fig 5, top panel). However, the absence of the metronome resulted in a significant increase of the variance in *early off* (t2) t(9) = -2.68, p = 0.025) and *late off* periods (t3) (t(9) = -3.0, p = 0.02). The average phase did not drift away from the entrained phase.

Similar to the parallel condition, the anti-phase was more difficult to perform and maintain in the orthogonal condition. Because two subjects were unable to do so, their data are not considered in Fig 5, second row. The remaining eight subjects followed the imposed anti-phase relationship during *metronome on* (μ = 185.3°, σ = 18.6°). Without this guidance (*early off*), the mean shifted away from the *metronome on* value (t(7) = -3.14, p = 0.016) and became significantly different from 180° (t(7) = 2.80, p = 0.024). This was accompanied by a significant increase in variance once the metronome was switched off in *early off* (t(7) = -2.4, p = 0.048) and *late off*(t(7) = -2.92, p = 0.022). The latter is again more prominently visible when including the initially excluded subjects (Fig 5, bottom panel).

### Comparison to different minimum cost models

#### Minimum energy

We used a minimum energy model (see methods) to estimate the most efficient relative phase between arm and whole body motion. For the parallel movement condition, this model predicted the optimal phase relationship around 170° (Fig 2B), which is close to anti-phase. Our experimental data, however, show that subjects prefer quite the opposite: subjects were most stable in the in-phase relationship. For the orthogonal condition, the model did not predict a clear optimal phase relationship, as the energy expenditure was relatively constant across the different phase angels (Fig 2B). Also, the experimental performance of the subjects showed no clear phase-preference, irrespective of whether the imposed relationship was in or anti-phase.

#### Minimum variance

We also tested a minimum endpoint variance model (see methods) to estimate the most efficient relative phase between arm and sled movement. For the parallel movement condition the minimum variance model predicted the optimal phase relationship to be around 160 degrees (Fig 2B), which is not in agreement with the data. For the orthogonal condition, the minimum variance model predicted no clear optimal solution. This is in line with the behavioral results, showing no clear phase tuning either.

## Discussion

In this study, we examined the emerging coordination between cyclic reaching movements and passive whole body motion. Phase relationships between arm and body motion were determined and evaluated based on two different minimum cost models. Our experimental results show a stable in-phase relationship when reaches are made between targets that are aligned parallel to the body motion. A striking observation was that, even when the phase relationship was initially entrained in anti-phase, subjects readily tended to drift back towards an in-phase relationship once the entraining metronome disappeared. This is in contrast with the predictions of both the energetic cost and the endpoint variance cost model, which both showed an optimum close to anti-phase. For the orthogonal reaches, subjects did not show a clear difference in stability for in-phase and anti-phase relationships. After removal of the entraining metronome, only the variance of the phase relationship increased but no phase transitions were observed. This is in line with both model predictions, which did also not show a clear optimal coordination phase.

Our paradigm differs from previous studies on reaching during whole body motion [1,3,5,12] by studying continuous arm-trunk coordination rather than the isolated control of a single arm and body movement. Because the reaches can always achieve the target, irrespective of the position of the body, there is a free choice in their initiation while the body moves.

The inertial forces imposed on the arm by the whole body motion were either assistive or resistive to the hand motion for the in-phase and anti-phase reaches parallel to the body. Therefore, the consistent phase convergence in the parallel condition suggests an inertial influence on the emergence of this phase relationship. The lack of a clear phase convergence in the orthogonal condition supports this notion, because the inertial forces due to body acceleration were of similar magnitude for the in-phase and anti-phase reaches. Remarkably however, the predicted optimal phase relationships for the parallel reaches were opposite to those observed experimentally, for both cost functions.

Why do our models fall short to explain the observed coordination pattern in the parallel condition? For the parallel condition, in-phase reaching required muscular forces that compensate for the inertial forces on top of the forces required to move the arm to the target. In contrast, when reaching in anti-phase, subjects could exploit the inertial forces and have their arm passively moved between the targets. As predicted by our model, the latter is the energetically and kinematic more efficient solution, but not what we found.

Previous studies using reach adaptation in static environments have also shown that energy is not always the most important factor that drives the movement [26–29]. Kistemaker and co-workers [27,28] showed that subjects, after they successfully learnt to reach through a force field along an energetic optimal path, do not choose this path in trials where they were free to choose how to move through the force field. The authors argue that the brain first solves kinematic redundancy and subsequently solves muscle redundancy by minimizing the energetic costs for the chosen trajectory. Applying this notion to the present findings, it could still be the case that energy is minimized, but only in a later stage and hence not the dominant factor that determines the phase relationship in continuous arm–trunk coordination.

The endpoint variance model could also not predict our experimental results. The model showed similar phase tuning as the energy model did. This suggests that there is another, maybe additional, cost that is minimized.

A possible candidate cost might relate to the stability of the movement. Note, we mean the stability of a mechanical system, here the arm, not the stability of a phase relationship. It has been shown that subjects increase the impedance of the limb in response to instability [24,26,30]. Impedance control offers more stability, but at the cost of higher energy consumption due to muscular co-contraction. Changes in impedance have been shown to suppress the effects of variability in the muscular forces on the kinematics of the movement [24,31]. The higher muscular forces, and the accompanied increase in impedance, needed to move the arm in-phase with the whole body motion may therefore be beneficial to the accuracy of the movement endpoints. Alternatively, subjects could have chosen to move in anti-phase, but in that case they would have needed to co-contract their muscles to increase the impedance and reduce their kinematic variability. However, this would also have diminished the passive movement of the limb due to the inertial forces and additional active torques would be required. So, following this reasoning, it might actually be that the anti-phase pattern is the energetically more demanding solution. For the orthogonal condition this trade-off between energy consumption and control of stability is less pronounced, as the magnitude of the inertial forces in both in-phase and anti-phase are very similar. For future work on this topic, EMG should be measured to monitor impedance control.

Furthermore, it has been shown, based on the variability of acceleration, that noise levels differ between concentric and eccentric contractions of muscle [32,33]. Concentric muscle contractions cost more energy than eccentric contractions, but seem to be accompanied by smaller fluctuations of acceleration. Could this difference in noise levels explain, perhaps partly, the preference for in-phase reaching in the parallel condition? In-phase reaches parallel to the body motion predominantly involve concentric contractions as the external torques are opposite the arm motion. In contrast, anti-phase reaches involve eccentric contractions as the external torques are in the same direction as the arm motion. Given the difference in variability associated with eccentric and concentric contractions, the optimal solution to the minimum variance problem may yield the in-phase coordination pattern.

We would like to point out that it is entirely plausible that our results are not necessarily best interpreted in terms of a cost function. The observed coordination behavior could also be driven by neural constraints. For example, gaze has been shown to influence hand movements, as if the hand is anchored to gaze [34,35]. If the eyes prefer an in-phase pattern when looking to the targets, for example because of vestibular influences on eye movements, the hand might be pushed into the same coordination pattern. Because we did not measure eye-movements, we could not address this hypothesis.

The existence of a preferred phase relationship has been described extensively for all kinds of rhythmic behavior, including interlimb coordination[36–39], between locomotion and respiration [40] and in juggling tasks [41]. Typically the preferred phase relationship, i.e. phase-locking, is thought to reflect the emergence of a stable dynamical system, in which the existing task dynamics (both in terms of biomechanical and neuronal constraints and dynamics) are exploited rather than cancelled out [41,42]. It remains elusive which biomechanical and neuronal constraints lead to the emergence of this pattern.

In conclusion, our study suggests that the brain compensates for the inertial forces when coordinating reach behavior and cyclic body motion. While the neural solution of this coordination is still unclear, predictive and impedance control processes could be considered important mechanisms to study in future work.

## References

[1] Bresciani J-P, Gauthier GM, Vercher J-L, Blouin J. On the nature of the vestibular control of arm-reaching movements during whole-body rotations. Exp Brain Res. 2005;164: 431–441. 10.1007/s00221-005-2263-4 15895218

[2] Moreau-Debord I, Martin CZ, Landry M, Green AM. Evidence for a reference frame transformation of vestibular signal contributions to voluntary reaching. J Neurophysiol. 2014;111: 1903–19. 10.1152/jn.00419.2013 24523527

[3] Pigeon P, Feldman AG. Compensatory arm–trunk coordination in pointing movements is preserved in the absence of visual feedback. Brain Res. 1998;802: 274–280. 10.1016/S0006-8993(98)00616-7 9748626

[4] Rosenbaum DA. Reaching while walking: reaching distance costs more than walking distance. Psychon Bull Rev. 2008;15: 1100–1104. 10.3758/PBR.15.6.1100 19001574

[5] Medendorp WP, Van Asselt S, Gielen CC a M. Pointing to remembered visual targets after active one-step self-displacements within reaching space. Exp Brain Res. 1999;125: 50–60. 10.1007/s002210050657 10100976

[6] Bresciani J-P, Blouin J, Popov K, Sarlegna F, Bourdin C, Vercher J-L, et al Vestibular signals contribute to the online control of goal-directed arm movements. Curr Psychol Cogn. 2002;21: 263–280.

[7] Cullen KE. The vestibular system: multimodal integration and encoding of self-motion for motor control. Trends Neurosci. 2012;35: 185–96. 10.1016/j.tins.2011.12.001 22245372 PMC4000483

[8] Sarwary AME, Selen LPJ, Medendorp WP. Vestibular benefits to task savings in motor adaptation. J Neurophysiol. 2013;110: 1269–77. 10.1152/jn.00914.2012 23785131 PMC3763157

[9] Todorov E, Jordan MI. Optimal feedback control as a theory of motor coordination. Nat Neurosci. 2002;5: 1226–35. 10.1038/nn963 12404008

[10] Prsa M, Jimenez-Rezende D, Blanke O. Inference of perceptual priors from path dynamics of passive self-motion. J Neurophysiol. 2015;113: 1400–1413. 10.1152/jn.00755.2014 25505114

[11] Bernstein N. The co-ordination and regulation of movements. Oxford: Pergamon Press; 1967.

[12] Ma S, Feldman AG. Two Functionally Different Synergies During Arm Reaching Movements Involving the Trunk. J Neurophysiol. 1995;73: 2120–2122. 7623104 10.1152/jn.1995.73.5.2120

[13] Pigeon P, Yahia L, Mitnitski AB, Feldman AG. Superposition of independent units of coordination during pointing movements involving the trunk with and without visual feedback. Exp Brain Res. 2000;131: 336–349. 10.1007/s002219900267 10789948

[14] Todorov E. Optimality principles in sensorimotor control. Nat Neurosci. 2004;7: 907–915. 10.1038/nn1309 15332089 PMC1488877

[15] Kelso JAS. The informational character of self-organized coordination dynamics. Hum Mov Sci. 1994;13: 393–413. 10.1016/0167-9457(94)90047-7

[16] Roerdink M, Ridderikhoff A, Peper CE, Beek PJ. Informational and neuromuscular contributions to anchoring in rhythmic wrist cycling. Ann Biomed Eng. 2013;41: 1726–1739. 10.1007/s10439-012-0680-7 23099793 PMC3701797

[17] Huys R, Daffertshofer A, Beek PJ. Learning to juggle: on the assembly of functional subsystems into a task-specific dynamical organization. Biol Cybern. 2003;88: 302–318. 10.1007/s00422-002-0379-1 12690489

[18] Ronsse R, Wei K, Sternad D. Optimal control of a hybrid rhythmic-discrete task: the bouncing ball revisited. J Neurophysiol. 2010;103: 2482–2493. 10.1152/jn.00600.2009 20130042 PMC2867585

[19] Alexander RM. A minimum energy cost hypothesis for human arm trajectories. Biol Cybern. 1997;76: 97–105. 9116080 10.1007/s004220050324

[20] Uno Y, Kawato M, Suzuki R. Formation and Control of Optimal Trajectory in Human Multijoint Arm Movement. Biol Cybern. 1989;61: 89–101. 2742921 10.1007/BF00204593

[21] Harris CM, Wolpert DM. Signal-dependent noise determines motor planning. Nature. 1998;394: 780–784. 9723616 10.1038/29528

[22] Scholz JP, Kelso JAS. A quantitative approach to understanding the formation and change of coordinated movement patterns. J Mot Behav. 1989;21: 122–144. 10.1080/00222895.1989.10735470 15132941

[23] Berens P. CircStat: A MATLAB Toolbox for Circular Statistics. J Stat Softw. 2009;31: 1–21.

[24] Selen LPJ, Franklin DW, Wolpert DM. Impedance control reduces instability that arises from motor noise. J Neurosci. 2009;29: 12606–12616. 10.1523/JNEUROSCI.2826-09.2009 19812335 PMC2784227

[25] Flash T, Hogan N. The Coordination of Arm Movements: Mathematical Model. J Neurosci. 1985;5: 1688–1703. 4020415 10.1523/JNEUROSCI.05-07-01688.1985 PMC6565116

[26] Balasubramanian R, Howe RD, Matsuoka Y. Task performance is prioritized over energy reduction. IEEE Trans Biomed Eng. 2009;56: 1310–1317. 10.1109/TBME.2008.2006683 19272896

[27] Kistemaker DA, Wong JD, Gribble PL. The central nervous system does not minimize energy cost in arm movements. J Neurophysiol. 2010;104: 2985–2994. 10.1152/jn.00483.2010 20884757

[28] Kistemaker DA, Wong JD, Gribble PL. The cost of moving optimally: kinematic path selection. J Neurophysiol. 2014;112: 1815–1824. 10.1152/jn.00291.2014 24944215 PMC4200004

[29] Thoroughman KA, Wang W, Tomov DN. Influence of viscous loads on motor planning. J Neurophysiol. 2007;98: 870–877. 10.1152/jn.01126.2006 17522176

[30] Franklin DW, So U, Kawato M, Milner TE. Impedance control balances stability with metabolically costly muscle activation. J Neurophysiol. 2004;92: 3097–3105. 10.1519/JSC.0b013e31823bc0a2 15201309

[31] Selen LPJ, Beek PJ, Van Dieën JH. Can co-activation reduce kinematic variability? A simulation study. Biol Cybern. 2005;93: 373–381. 10.1007/s00422-005-0015-y 16249892

[32] Duchateau J, Enoka RM. Neural control of shortening and lengthening contractions : influence of task constraints. J Physiol. 2008;586: 5853–5864. 10.1113/jphysiol.2008.160747 18955381 PMC2655422

[33] Christou EA, Shinohara M, Enoka RM. Fluctuations in acceleration during voluntary contractions lead to greater impairment of movement accuracy in old adults. J Appl Physiol. 2003;95: 373–384. 12651861 10.1152/japplphysiol.00060.2003

[34] Neggers SFW, Bekkering H. Ocular gaze is anchored to the target of an ongoing pointing movement. J Neurophysiol. 2000;83: 639–651. 10669480 10.1152/jn.2000.83.2.639

[35] Neggers SFW, Bekkering H. Integration of visual and somatosensory target information in goal-directed eye and arm movements. Exp Brain Res. 1999;125: 97–107. 10.1007/s002210050663 10100982

[36] Kelso JAS. Phase transitions and critical behavior in human bimanual coordination. Am J Psysiology Regul Integr Comp. 1984; 10.1152/ajpregu.1984.246.6.R1000 6742155

[37] Ridderikhoff A, Peper CE, Beek PJ. Unraveling interlimb interactions underlying bimanual coordination. J Neurophysiol. 2005;94: 3112–3125. 10.1152/jn.01077.2004 16000517

[38] Ridderikhoff A, Peper CE, Beek PJ. Bilateral phase entrainment by movement-elicited afference contributes equally to the stability of in-phase and antiphase coordination. Neurosci Lett. 2006;399: 71–75. 10.1016/j.neulet.2006.01.026 16472912

[39] Swinnen SP, Dounskaia N, Verschueren S, Serrien DJ, Daelman A. Relative phase destabilization during interlimb coordination: the disruptive role of kinesthetic afferences induced by passive movement. Exp Brain Res. 1995;105: 439–454. 7498398 10.1007/BF00233044

[40] Daffertshofer A, Huys R, Beek PJ. Dynamical coupling between locomotion and respiration. Biol Cybern. 2004;90: 157–164. 10.1007/s00422-004-0462-x 15052479

[41] Schaal S, Atkeson CG, Sternad D. One-Handed Juggling: A Dynamical Approach to a Rhythmic Movement Task. J Mot Behav. 1996;28: 165–183. 10.1080/00222895.1996.9941743 12529218

[42] Sternad D, Duarte M, Katsumata H, Schaal S. Dynamics of a bouncing ball in human performance. Phys Rev E. 2000;63: 0119021–0119028. 10.1103/PhysRevE.63.011902 11304282

